# A Case Report of Fahr’s Disease and Its Clinical Heterogeneity

**DOI:** 10.7759/cureus.51065

**Published:** 2023-12-25

**Authors:** Shraddha Adhikari, Archana Bhate, Smita Patil, Mihit Kalawatia, Ravi Sangoi, Amisha Palande, Parijat Kamble, Gaurav Mittal

**Affiliations:** 1 General Medicine, Dr. D. Y. Patil Medical College, Navi Mumbai, IND; 2 Medicine, Rajarshee Chatrapati Shahu Maharaj Government Medical College, Kolhapur, IND; 3 Internal Medicine, Punyashlok Ahilyadevi Holkar Government Medical College and General Hospital, Baramati, Baramati, IND; 4 Physiology, Terna Medical College, Mumbai, IND; 5 General Medicine, Terna Medical College, Mumbai, IND; 6 Research and Development, Rotaract Club of Indian Medicos, Mumbai, IND; 7 Research, Students Network Organization, Mumbai, IND; 8 Internal Medicine, Mahatma Gandhi Institute of Medical Sciences, Wardha, IND

**Keywords:** calcium metabolism, seizure, basal ganglia disease, cerebral cortex, neurological diseases

## Abstract

Fahr's disease is an exceptionally rare and complex neurological disorder characterized by abnormal calcium deposition in the basal ganglia and cerebral cortex. This case report presents a 27-year-old female with Fahr's disease, showcasing the striking clinical diversity and challenging diagnostic landscape associated with this condition. Despite its rarity, Fahr's disease can have a profound impact on patients, manifesting as a spectrum of neurological symptoms, cognitive deficits, and motor impairment. Recent advancements in research have illuminated genetic aspects, offering potential avenues for enhanced diagnostic accuracy and therapeutic interventions. Treatment for Fahr's disease remains primarily supportive, targeting neuropsychiatric symptoms and seizure prophylaxis. Our case highlights the unique presentation of a young female patient with Fahr's disease, challenging conventional demographic profiles and emphasizing the need for individualized patient assessments. The utilization of non-contrast CT scans in diagnosis underscores the importance of appropriate imaging techniques, given the diverse clinical manifestations of this condition. This case adds to the growing understanding of Fahr's disease, emphasizing its clinical heterogeneity and advancing the quest for tailored approaches to diagnosis and intervention.

## Introduction

Fahr's disease, also known as idiopathic basal ganglia calcification, is an exceptionally rare and enigmatic neurological disorder characterized by the abnormal deposition of calcium in the basal ganglia and cerebral cortex of the brain [1]. This condition, although infrequently encountered in clinical practice, poses considerable challenges due to its varied clinical presentations and the absence of a well-defined pathophysiological mechanism. The patient commonly presents with chief complaints of anxiety, giddiness, aggressive behavior, insomnia, and lack of concentration [2]. The significance of Fahr's disease lies not only in its rarity but also in the profound impact it can have on patients' lives, leading to a spectrum of neurological symptoms, cognitive deficits, and motor impairment. Amidst the relatively rare occurrence of Fahr's disease, a growing body of literature is dedicated to understanding its etiology, diagnostic criteria, and therapeutic approaches [2,3]. Recent advancements in research have illuminated the genetic underpinnings of Fahr's disease, illuminating pathways to improved diagnostic precision and potential interventions [4]. There is currently no curative treatment for Fahr’s disease; therefore, treatment is supportive and focused on improving neuropsychiatric symptoms and seizure prophylaxis. We demonstrate a case of Fahr’s disease in a 27-year-old female who presented with generalized weakness and psychiatric symptoms. She was incidentally found to have extensive bilateral basal ganglia calcifications in the setting of normal lab values.

## Case presentation

A 27-year-old female presented to our outpatient department with a chief complaint of persistent headaches lasting for the past six months. The headaches were not associated with vomiting or visual disturbances. Upon further inquiry, the patient reported a history of recurrent episodes of giddiness and seizures over the past few months. Additionally, she described experiencing intermittent bouts of aggressive behavior and anxiety. The patient's aggressive outbursts occurred less frequently, about once a week, and varied in severity from verbal outbursts to physical attacks. These episodes are triggered by specific events or situations or even occur without apparent provocation. As for her anxiety, it was a constant presence, mild in nature but significantly interfering with daily life. She also experienced some symptoms such as restlessness, irritability, difficulty concentrating, insomnia, and excessive worry. Giddiness episodes were brief, lasting only a few seconds, while seizures were less frequent but notable in the patient's clinical profile. The patient had a past medical history notable for mild asthma, well-controlled with inhalers, with no recent exacerbations. She had no significant history of major illnesses or surgeries. Her psychiatric history was significant for a diagnosed generalized anxiety disorder at the age of 26, for which she had been on intermittent therapy, including cognitive-behavioral therapy and Selective Serotonin Reuptake Inhibitors (SSRIs), with moderate success. There was no documented history of major depressive disorder, bipolar disorder, or schizophrenia. She did not experience any notable physical trauma or require hospitalization or medical intervention for trauma. Regarding substance use, the patient denied illicit drug use and tobacco consumption. She admitted to occasional alcohol use, which has not been linked to her current health concerns. Her regular medication intake before the present symptoms included only her asthma inhalers and SSRIs for anxiety management.

Upon initial examination, the patient appeared to be in stable condition, with normal vital signs. Notably, there were no motor or sensory deficits detected during the clinical assessment. Laboratory investigations, including a complete hemogram screening, were conducted to sort out differential diagnoses. These tests included measuring blood and urine heavy metal levels to rule out brain calcifications due to heavy metal exposure. Additionally, levels of calcium, phosphorus, magnesium, alkaline phosphatase (ALP), calcitonin, vitamin D, and parathyroid hormone (PTH) were assessed to evaluate the functioning of the parathyroid gland and the regulation of calcium metabolism. Furthermore, cerebrospinal fluid analysis was recommended to check for the presence of bacteria, viruses, or parasites that could be causing or contributing to brain calcifications. All these results were found within normal reference ranges. Testing for infectious diseases, including toxoplasmosis, yielded inconclusive results.

**Figure 1:**
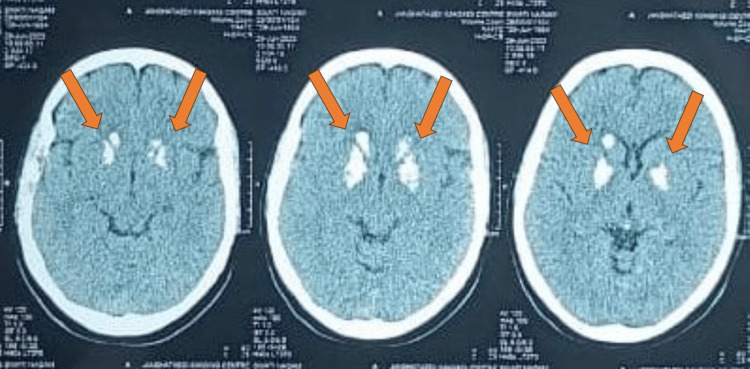
A non-contrast CT Scan of the head The arrows depict calcification in the basal ganglia.

**Figure 2:**
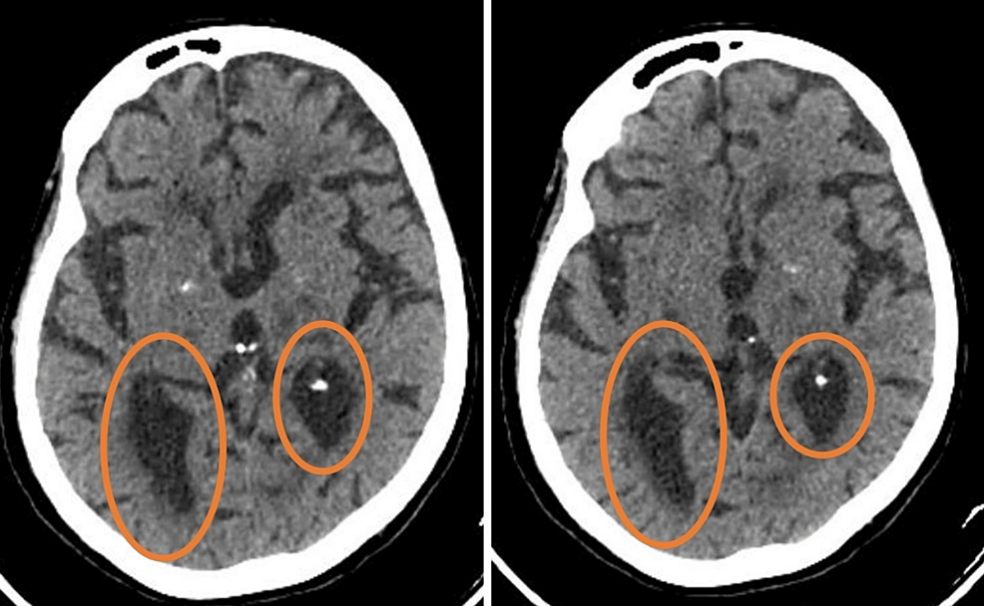
A non-contrast CT Scan of the head The marked circles show basal ganglia calcification.

A comprehensive neurologic examination was conducted, revealing no abnormalities, indicating normal motor and sensory functions. The patient also reported intermittent episodes of aggressive behavior and anxiety. Given the patient's clinical presentation and neurological findings, a non-contrast CT (NCCT) scan of the head was performed, which has been shown in Figure 1 and Figure 2. The imaging results revealed extensive bilateral basal ganglia calcifications. These radiological findings raised suspicion of Fahr's disease as a potential diagnosis. To differentiate this case from other conditions with similar radiological findings, such as Fahr's syndrome, idiopathic basal ganglia calcification (IBGC), and primary familial brain calcification (PFBC), a comprehensive workup was conducted. Laboratory investigations were performed to rule out hypoparathyroidism, hypothyroidism, heavy metal poisoning, infections, and other neurodegenerative disorders. The exclusion of known metabolic, toxic, infectious, and endocrinologic causes led to the consideration of IBGC or Fahr's syndrome. PFBC, which is a familial form of IBGC, was also considered and excluded due to the absence of a relevant family history and no association with known calcium, phosphorus, or copper metabolism abnormalities. Genetic mutations associated with PFBC, such as solute carrier family 20 member 2 gene (SLC20A2) and platelet-derived growth factor subunit B (PDGFB), were investigated and found to be absent, further supporting the diagnosis of Fahr's disease.

After establishing the diagnosis of Fahr's disease, the patient's management plan was initiated, focusing on symptomatic relief and eradicating causative factors. This included calcitriol supplementation and antiepileptic therapy with Levetiracetam 500mg, given twice daily, which was prescribed for seizure control. Pain management for headaches was approached with the administration of non-steroidal anti-inflammatory drugs (NSAIDs). SSRIs were prescribed, to be taken once daily, for aggressive behavior and anxiety. Neuroleptics were avoided to prevent exacerbation of extrapyramidal symptoms. The patient's response to this treatment regimen was monitored closely. There was a marked improvement in her symptoms following the initiation of the treatment. The frequency of seizures and the intensity of headaches significantly reduced, and there was a noticeable decrease in episodes of aggressive behavior and anxiety. This improvement greatly enhanced the patient's quality of life and daily functioning. Follow-up consultations were scheduled to assess the effectiveness of the treatment and to monitor the patient's overall condition. At these follow-up visits, the patient showed continued improvement and stability in her condition, indicating a positive response to the treatment regimen.

## Discussion

Fahr's disease, a rare neurodegenerative disorder characterized by extensive basal ganglia and cerebral calcifications, has garnered attention for its diverse clinical presentations and diagnostic challenges. Again, although Fahr's syndrome and Fahr's disease share certain clinical similarities, such as neurological and psychiatric manifestations, they remain distinct with clear differences in terms of their etiology, the location of calcifications, and treatment approaches. Fahr's syndrome is diagnosed when basal ganglia calcifications are identified as secondary to known causes and associated clinical presentations align with the condition. This distinction is crucial, as the calcifications in Fahr's syndrome are typically a consequence of other medical conditions, such as metabolic disorders or infections. Therefore, identifying the underlying cause of the calcifications is a fundamental aspect of diagnosing Fahr's syndrome [5]. In contrast, Fahr's disease is diagnosed based on clinical presentation, imaging evidence, and associated family history after excluding other intracranial calcification causes. This condition is considered primary, and the calcifications are a standard feature of the disease itself. Fahr's disease is often inherited in an autosomal dominant manner, and several gene mutations have been linked to it, including PDGFB, platelet-derived growth factor receptor-beta (PDGFRB), SLC20A2, and xenotropic and polytropic retrovirus receptor 1 (XPR1) as explained in the study by Ramos et al. [6].

An early-onset variant of Fahr's disease has been identified in a study by Aghemo et al. [7], primarily observed in individuals aged 40 or in their early 30s. This variant displays a unique clinical trajectory where psychiatric symptoms precede the onset of movement disorders. Our case presents a patient in her late twenties who aligns with the profile of this early-onset variant. However, it's crucial to acknowledge that the patient's clinical history deviates from the expected symptom timeline of this variant. In our case, the patient initially exhibited recurrent episodes of aggressive behavior and anxiety, which are standard psychiatric symptoms commonly associated with Fahr's disease and are also noted in the early variant described in the mentioned study [7]. Nevertheless, conspicuously absent in this case were gait abnormalities, which are typically recognized as a later-stage manifestation of the disease. This divergence underscores the notable clinical heterogeneity that characterizes Fahr's disease, demonstrating that the early variant may not uniformly exhibit all clinical presentations.

Furthermore, our case challenges the traditional demographic profile of Fahr's disease. It is commonly reported that Fahr's disease is more frequently diagnosed in males and primarily manifests after the age of 30, as described in a study by Kotan and Aygul [8]. However, the case of a 27-year-old female patient in our report challenges these conventional demographics and age-related expectations. The earlier onset of symptoms in our patient, who is in her late twenties, underscores the considerable variability in the age of presentation associated with Fahr's disease. This observation emphasizes the need for healthcare providers to maintain a high index of suspicion for Fahr's disease, even among younger patients who exhibit relevant clinical features. It also highlights the importance of individualized patient assessments and a comprehensive diagnostic approach tailored to the specific clinical characteristics of each case.

The utilization of an NCCT scan in the diagnostic process of Fahr's disease, as illustrated in the presented case, aligns with the findings of Savino et al. [9]. NCCT has been recognized as the most sensitive imaging modality for detecting the characteristic bilateral basal ganglia calcifications. The case report underscores the significance of selecting the appropriate imaging technique, such as NCCT, when Fahr's disease is suspected even though there is no movement disability. The ability of NCCT to precisely identify areas of calcification in the basal ganglia enhances the accuracy of diagnosis.

An NCCT scan is confirmatory for Fahr's disease as it can detect bilateral basal ganglia calcification, which is a characteristic feature of the disease [1,2]. Fahr's disease is differentiated from other causes of basal ganglia calcification by considering the clinical correlation with radiological findings and a calcium metabolism panel [8]. Other causes of basal ganglia calcification include Fahr's syndrome, which is characterized by bilateral striato-pallido-dentate calcinosis and can present with similar clinical features as Fahr's disease [9]. Additionally, IBGC or Fahr's disease can be caused by genetic mutations, such as mutations in the SLC20A2 gene, which can be identified through genetic testing. Therefore, an NCCT scan is an important tool in confirming the diagnosis of Fahr's disease and differentiating it from other causes of basal ganglia calcification, while genetic testing can further aid in identifying specific genetic mutations associated with the disease. This is particularly crucial as Fahr's disease often presents with a diverse range of clinical symptoms and can mimic other neurological conditions.

Autoimmune polyendocrinopathy-candidiasis-ectodermal dystrophy (APECED) syndrome, is characterized by a high frequency of Fahr's syndrome due to autoimmune parathyroidism. APECED is a rare syndrome inherited in an autosomal recessive manner, resulting from mutations in the autoimmune regulator (AIRE) gene. The syndrome is characterized by three main clinical components: mucocutaneous candidiasis, hypoparathyroidism, and primary adrenal insufficiency. Beyond these common symptoms, various other manifestations, including gastrointestinal symptoms, have been documented [10].

In our case, we observed and documented the occurrence of headaches, giddiness, seizures, and fluctuations in mood, including episodes of aggressive behavior and anxiety. These findings closely parallel the neuropsychiatric and somatic symptomatology highlighted in the broader scientific discourse on Fahr's disease [11]. This concurrence underscores the diverse clinical presentation of Fahr's disease, with our case lending further support to the notion that neurological symptoms can manifest across a wide spectrum in affected individuals.

However, it is essential to acknowledge the unique aspect of our case. Unlike some instances described in the literature [7], our patient did not exhibit movement disorders or other neuropsychiatric symptoms such as hallucinations, delusions, or mood disorders like depression or bipolar disorder. This divergence highlights the clinical heterogeneity that characterizes Fahr's disease. It emphasizes the intriguing reality that patients with Fahr's disease may display a wide array of symptoms, and the specific clinical presentation can indeed vary significantly from case to case.

## Conclusions

Fahr's disease, a rare neurological disorder with calcifications in the basal ganglia and cerebral region, shows that every case is unique. Our case report vividly illustrates the remarkable variability in clinical presentations, challenging preconceived notions and traditional demographic profiles associated with this condition. Fahr's disease, as our case exemplifies, defies a one-size-fits-all approach, emphasizing the need for individualized assessments and tailored diagnostic strategies. As we go deeper into the intricacies of this disorder, we continue to uncover the rich tapestry of symptoms it can weave, underscoring its clinical heterogeneity. With each case offering new insights, we inch closer to a more comprehensive understanding of Fahr's disease, ultimately paving the way for improved diagnostic precision and customized interventions to address the diverse challenges it presents to patients and healthcare providers.
